# Prospective analysis of 30-day postoperative adverse events in skull base surgery: insights into risk factors and mitigation strategies from a neurosurgical tertiary care center

**DOI:** 10.1007/s10143-025-03944-w

**Published:** 2025-11-20

**Authors:** M. Grutza, H. Bächler, D. Haux, P. Lenga, B. Suchorska, M. Scherer, S. Krieg, J. Walter, P. Dao Trong

**Affiliations:** 1 Department of Neurosurgery, Heidelberg University Hospital, Im Neuenheimer Feld 400, Heidelberg, 69120 Germany; 2 Medical Faculty of Heidelberg University, Heidelberg, Germany

**Keywords:** Skull base surgery, Adverse events, Clavien–Dindo classification, ASA score, Retrosigmoid craniotomy, Infratentorial

## Abstract

Introduction Skull base surgery presents significant challenges due to the complex anatomy and proximity of tumors to critical neurovascular structures. While advancements in surgical techniques have improved outcomes, the risk of postoperative adverse events (AEs) remains substantial. This study provides a prospective analysis of AEs and associated risk factors in skull base surgery, leveraging data from a high-volume tertiary neurosurgical center. The analysis focuses on tumor location, surgical craniotomies, and patient-specific factors to identify predictors of complications and guide risk mitigation strategies. Methods Between January 2022 and December 2023, 236 adult patients undergoing skull base surgery were prospectively enrolled. AEs—defined as any complication occurring within 30 days postoperatively—were systematically documented. Data collection included patient demographics, tumor characteristics, surgical craniotomies, intraoperative findings, and postoperative outcomes to identify risk factors for AEs. Statistical analyses were performed to assess associations between these variables and postoperative complications. Results The study cohort had a mean age of 56.8 ± 12.7 years, with tumor distribution supratentorial (55.1%) and infratentorial (44.9%). The most frequently utilized surgical craniotomies were retrosigmoid (40.3%), pterional (39.4%), and later lateral supraorbital (6.4%). Overall, 28.8% of patients experienced AEs, with 22.5% neurosurgical (e.g., new-onset cranial nerve deficits) and 8.5% non-neurosurgical (e.g., thromboembolic events, infections). Older age and higher ASA scores (p = 0.01) were significant predictors of non-neurosurgical AEs. Revision surgery was required in 6.8% of cases. Infratentorial tumor location and prolonged operative times were strongly associated with an increased risk of surgical complications (p = 0.001), while the retrosigmoid craniotomy was a key risk factor for both neurosurgical AEs and revision surgeries (p = 0.001). ROC analysis showed that combining age and ASA score improved prediction of non-neurosurgical AEs (combined AUC = 0.78 vs. age AUC = 0.70; ASA AUC = 0.72). Conclusion Our findings highlight critical and actionable risk factors influencing neurosurgical outcomes. We demonstrate that infratentorial tumor location and prolonged surgical duration significantly increase the likelihood of surgery-related adverse events, with the retrosigmoid craniotomy particularly elevating these risks. Notably, advanced age and higher ASA scores robustly predict non–surgery-related complications, with a combined predictive accuracy superior to each factor individually. These insights underscore the importance of meticulous preoperative risk assessment and tailored surgical strategies, enabling clinicians to proactively manage high-risk patients and improve postoperative outcomes.

## Introduction

Skull base surgery remains among the most challenging domains in neurosurgery due to the region’s intricate anatomy and its proximity to critical neurovascular structures, including the brainstem, cranial nerves, and major blood vessels. Conventionally, this complex anatomical landscape is divided into the anterior, middle, and posterior cranial fossae, each harboring distinct pathologies and necessitating tailored surgical craniotomies for optimal tumor resection [1, 2]. In the anterior and middle skull base, traditional transcranial routes—pterional, bifrontal, and supraorbital craniotomies—have long served as mainstays, particularly for resecting meningiomas at the tuberculum sellae and planum sphenoidale. Over the past few decades, however, advances in endoscopic technology have propelled endonasal endoscopic-assisted (EEA) procedures to the forefront for lesions with infrachiasmatic extension. By offering enhanced visualization and reduced surgical trauma, EEA approaches can minimize injury to the optic apparatus and preserve critical blood supply [3, 4]. Meanwhile, tumors in the posterior skull base are commonly accessed via more traditional pathways, such as suboccipital or retrosigmoid craniotomies [5].

Notwithstanding these technical evolutions, skull base surgery remains inherently high-risk. The delicate anatomy and complexity of surgical corridors contribute to a host of potential postoperative complications, including cranial nerve deficits, cerebrospinal fluid (CSF) leaks, vascular injury, and infections [3, 6–8]. Moreover, each surgical approach carries specific benefits and limitations that may vary according to tumor size, location, and degree of infiltration into surrounding structures. Recent developments—such as intraoperative navigation systems and neuromonitoring—have helped reduce some of these risks [9, 10]. Yet adverse events (AEs) persist, underscoring the need for consistent documentation and continuous quality improvement.

Morbidity and mortality conferences (MMCs) have emerged as crucial forums for identifying, classifying, and preventing repeated AEs [11]. Multiple classification systems now exist to standardize the evaluation of surgical complications, with the overarching goal of enhancing patient outcomes [11–13]. Given the high stakes of skull base surgery—where complications can result in prolonged hospital stays, unplanned readmissions, and lasting neurological deficits [9, 14, 15]—robust and reliable reporting of AEs is especially critical. Despite a breadth of retrospective studies detailing complications in skull base surgery, most rely on relatively small patient populations and may underrepresent the true scope of risks. Although prospective data on adverse events exist for spinal and general cranial procedures [16–18], there remains a clear gap in similarly comprehensive analyses for skull base interventions. This study seeks to fill that gap by presenting a prospective, 30-day postoperative evaluation of AEs in skull base surgery at a high-volume neurosurgical tertiary care center.

Through the identification and characterization of these AEs and their associated risk factors, this work aims to refine current quality metrics and develop targeted strategies to reduce complication rates.

## Methods

### Study design, inclusion and exclusion criteria

This study was conducted as a prospective investigation at a tertiary care hospital from January 2022 to December 2023. The research protocol was approved by the institutional ethics committee (reference number S-425/2022) and adhered to the ethical principles outlined in the Declaration of Helsinki. Following the approach described by Dao Trong et al. and Lenga et al. [16, 18], the research team included 15 board-certified neurosurgeons and 18 neurosurgical residents, all of whom were responsible for the meticulous collection and updating of patient data in a dedicated database.

At the time of discharge, patients were provided with a form to report any postoperative adverse events (POPAEs), which was completed by the attending physician. These forms were reviewed by a senior neurosurgeon to ensure accuracy before being entered into the database. In cases where patients were readmitted within 30 days after surgery, the healthcare team received an automatic notification. Complex cases were presented and discussed during multidisciplinary morbidity and mortality conferences (MMC), which involved the entire neurosurgical staff.

This analysis specifically included adult patients with skull base tumors. Pediatric cases were excluded, as were patients with intracranial masses located outside the skull base. Tumors in the sellar region, such as a history of pituitary adenomas or craniopharyngiomas, were excluded from the study. Patients with tumors in the skull base were categorized into two groups: those with tumors in the anterior or middle skull base, which were classified as supratentorial masses, and those with tumors in the posterior cranial fossa, classified as infratentorial tumors. Additionally, the type of surgical craniotomy utilized in each case and patient positioning were included in the analysis.

### Classification of adverse events (AEs)

Adverse events (AEs) were categorized into surgery related -and not surgery related AEs. For surgery related AEs the following groups were defined: wound complications, postoperative infections, cerebrospinal fluid (CSF) leaks, new neurological deficits, postoperative hemorrhage, and failure to achieve surgical objectives. Elective surgery was defined as procedures scheduled at least one day in advance, while non-elective surgeries included emergency procedures and surgeries requiring revision. For the analysis of non-surgery-related AEs, patients were categorized into the following groups: pneumonia, thromboembolic event, electrolyte disorder, delirium, renal insufficiency, urinary tract infection, and others.

The following definitions were used to categorize specific surgery related adverse events:

- **Wound event**: Any complication related to wound healing, including both superficial and deep issues, as well as infections.
- **Postoperative infections**: Defined as the development of meningitis, abscesses, or empyema following surgery.
- **CSF fistula**: Any leakage of cerebrospinal fluid, either internal or external, including cases of rhinoliquorrhea.
- **Implant malfunction/CSF shunt dysfunction**: This category includes any dysfunction of CSF shunts, such as valve malfunction, mechanical obstruction, or catheter occlusion.
- **Malpositioning of implanted materials**: Refers to the incorrect placement of devices such as CSF catheters, pedicle screws, rods, or intervertebral cages.
- **New neurological deficit**: Describes any new neurological impairment that emerged after surgery, or the worsening of pre-existing deficits.
- **Rebleeding**: Refers to any bleeding in the resection cavity, subdural space, or surrounding soft tissues, resulting in new neurological deficits or requiring additional surgical intervention.
- **Surgical goal not achieved**: Refers to situations where the predefined objectives of surgery were not met, such as incomplete tumor resections when complete tumor resection was targeted.
- **Mortality**: Defined as death from any cause occurring within 30 days following surgery.
- **Electrolyte disturbance**: A newly developed electrolyte disturbance that can anatomically be explained by the surgeries in the area of the hypothalamic-pituitary axis.

To evaluate the severity of individual adverse events, the Clavien-Dindo classification system was used [11, 16].

### Statistical analysis

Descriptive statistics, including frequencies, percentages, means, and standard deviations, were used to summarize the data. To compare baseline characteristics, tumor location, surgical craniotomy, and adverse events (AEs) between groups, statistical tests such as independent t-tests and chi-squared tests were applied. A significance level of *p* ≤ 0.05 was considered statistically significant for all comparisons. We performed univariable logistic regression analyses to assess potential predictors of (I) surgery-related adverse events (AEs), (II) revision surgery, and (III) non-surgery-related AEs. Variables with clinical relevance or *p* < 0.10 in univariable analyses were entered into multivariable logistic regression models. Age and tumor diameter were modeled as continuous variables, ASA class as categorical (levels I–IV). Predicted probabilities from the final multivariable model were used to generate receiver operating characteristic (ROC) curves and to calculate the area under the curve (AUC). AUCs for single predictors (age, ASA) and the combined model (age + ASA) are reported descriptively; no formal statistical comparison between AUCs was performed. No missing data occurred in the analyzed cohort. All analyses were conducted in SPSS version 24 (IBM Corp., Armonk, NY, USA).

## Results

### Patient demographics and baseline characteristics

A total of 236 patients with skull base tumors were included in the study, of whom 65 (27.5%) were male and 171 (72.5%) were female. The mean patient age was 56.8 ± 12.7 years; 64 (27.1%) of these patients were aged 65 years or older. The average weight was 76.4 ± 18.2 kg, and the average height was 169 ± 9.3 cm, resulting in a mean body mass index (BMI) of 26.7 ± 5.6. A history of nicotine use was reported by 43 patients (18.2%). A detailed overview of patient demographics is provided in Table 1.

**Table 1:** Patient and tumor related characteristics

No. of patients	236
Age, years (mean, SD)	56.8 (12.7)
Age *≥ 65 years* (n, %)	64 (27.1)
Sex (n, %)	
Male	65 (27.5)
Female	171 (72.5)
Weight (in kg, mean, SD)	76.4 (18.2)
Height (in cm, mean, SD, cm)	169 (9.3)
BMI (mean, SD)	26.7 (5.6)
Smoking (n, %)	43 (18.2)
ASA – Score (n, %)	227
1	15 (6.4)
2	159 (67.4)
3	51 (21.6)
4	2 (0.8)
Tumor diameter (mean, SD, cm)	2.8 (1.4)
Histology (n, %)	
Meningeoma WHO Grade 1	172 (72.9)
Meningeoma WHO Grade 2	4 (1.7)
Neurinoma WHO Grade 1	55 (23.3)
Chordoma	2 (0.8)
Chrondrosarcoma	3 (1.3)
Tumor Localization (n, %)	
Supratentorial	130 (55.1)
Infratentorial	106 (44.9)
Patient Positioning in infratentorial Localization (n, %)	106
Semi Sitting	79 (74.5)
Park Bench	23 (21.7)
Concorde	4 (3.8)

Abbreviations: *ASA class* American Society of Anesthesiologists classification, *BMI* Body Mass Index

### Tumor entities and surgical characteristics

**Fig. 1:**
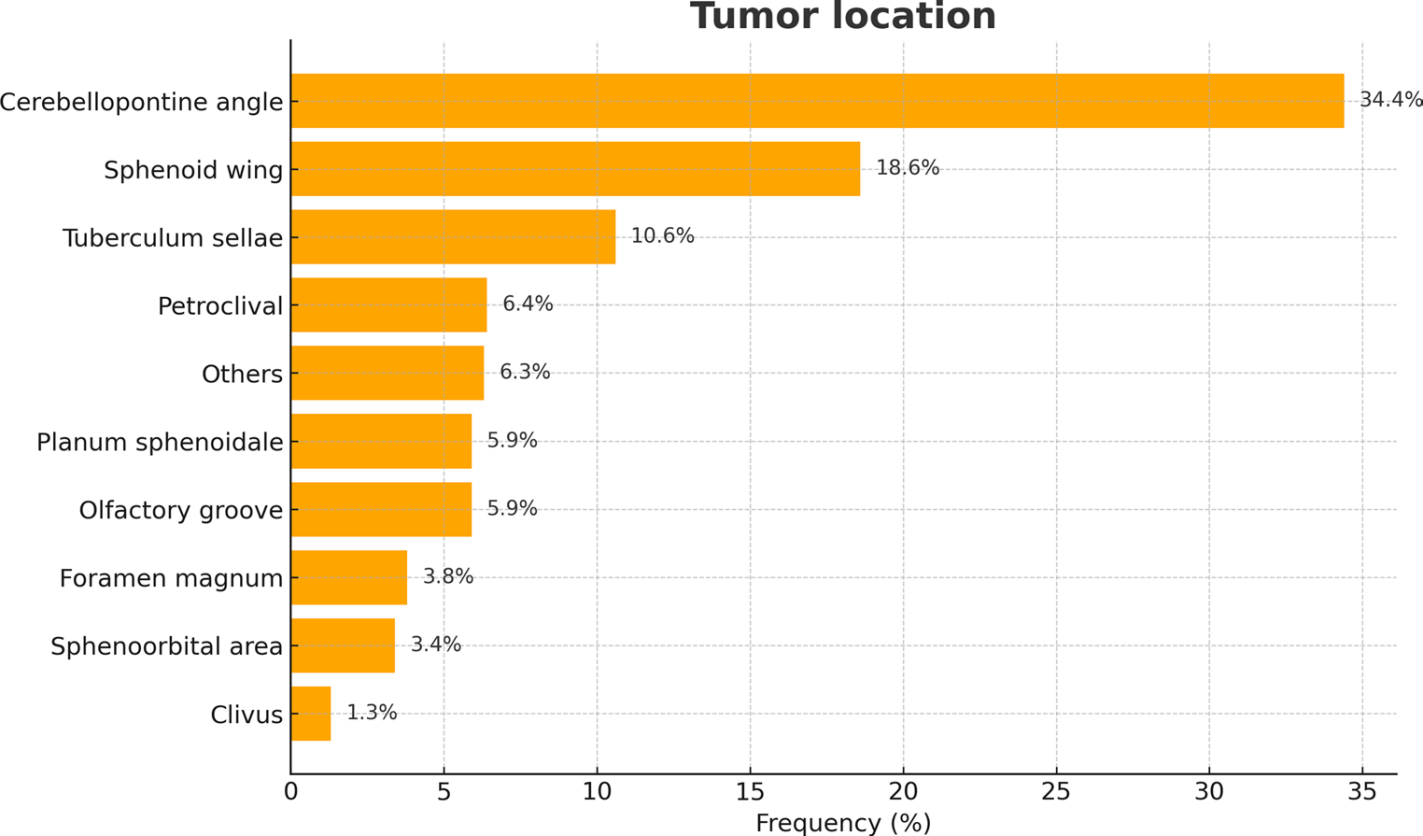
Tumor location

Of the 236 patients, 130 (55.1%) had tumors in the supratentorial region, while the remaining 106 (44.9%) had infratentorial tumors. The average tumor diameter across both groups was 2.8 ± 1.4 cm. In the supratentorial cohort, the most common tumor localization was sphenoid wing (*n* = 44, 18.6%), tuberculum sellae (*n* = 25, 10.6%), planum sphenoidale (*n* = 14, 5.9%), and olfactory groove (*n* = 14, 5.9%). Less frequent were spheno-orbital area (*n* = 8, 3.4%), and other supratentorial skull base tumors (*n* = 25, 10.6%). Among the infratentorial location, cerebellopontine angle (*n* = 81, 34.4%) was most prevalent, followed by petroclival (*n* = 15, 6.4%) foramen magnum (*n* = 9, 3.8%) and in the area of the clivus (*n* = 3, 1.2%). In cases of infratentorial tumor location, 79 patients (74.5%) were operated in the semi sitting position, 23 patients (21.7%) in the park bench position, and 4 patients (3.8%) in the concorde position. Based on the histological analysis of the resected tissue, 172 patients (72.9%) had a WHO grade 1 meningioma, 4 patients (1.7%) had a WHO grade 2 meningioma, 55 patients (23.3%) had a WHO grade 1 neurinoma, 2 patients (0.8%) had a chordoma, and 3 patients (1.3%) had a chondrosarcoma. The distribution of skull base tumors is summarized in Fig. 1.

**Fig. 2:**
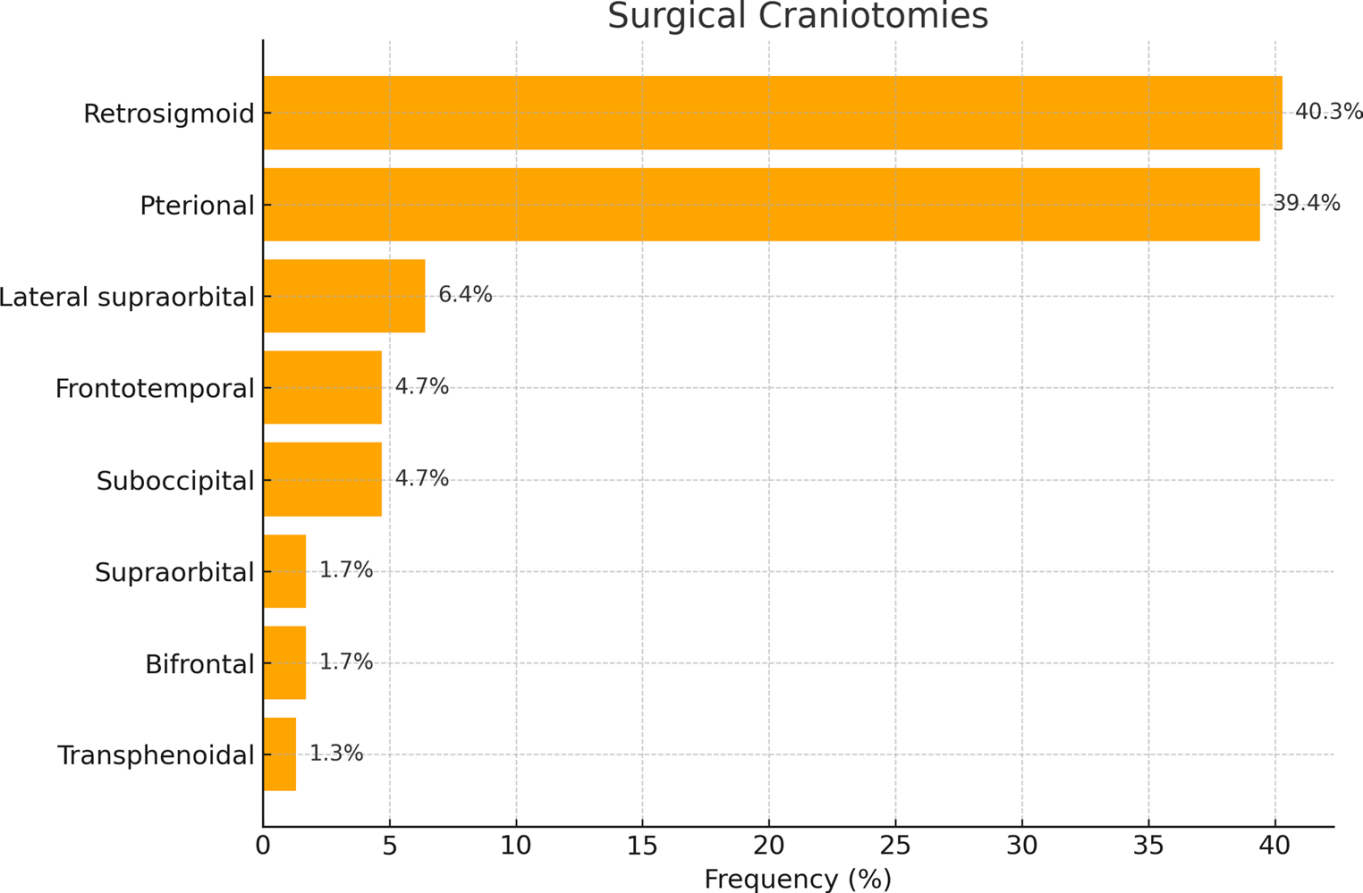
Surgical Craniotomies to the skull base

The mean duration of surgery was 284.4 ± 142.7 min. Surgical craniotomies included retrosigmoid (*n* = 95, 40.3%), pterional (*n* = 93, 39.4%), lateral supraorbital (LSO) (*n* = 15, 6.4%), frontotemporal (*n* = 11, 4.7%), suboccipital (*n* = 11, 4.7%), supraorbital (*n* = 4, 1.7%), bifrontal (*n* = 4, 1.7%) and transsphenoidal (*n* = 3, 1.3%). Surgical craniotomies to the skull base are summarized in Fig. 2.

## Occurrence of adverse events

**Fig. 3:**
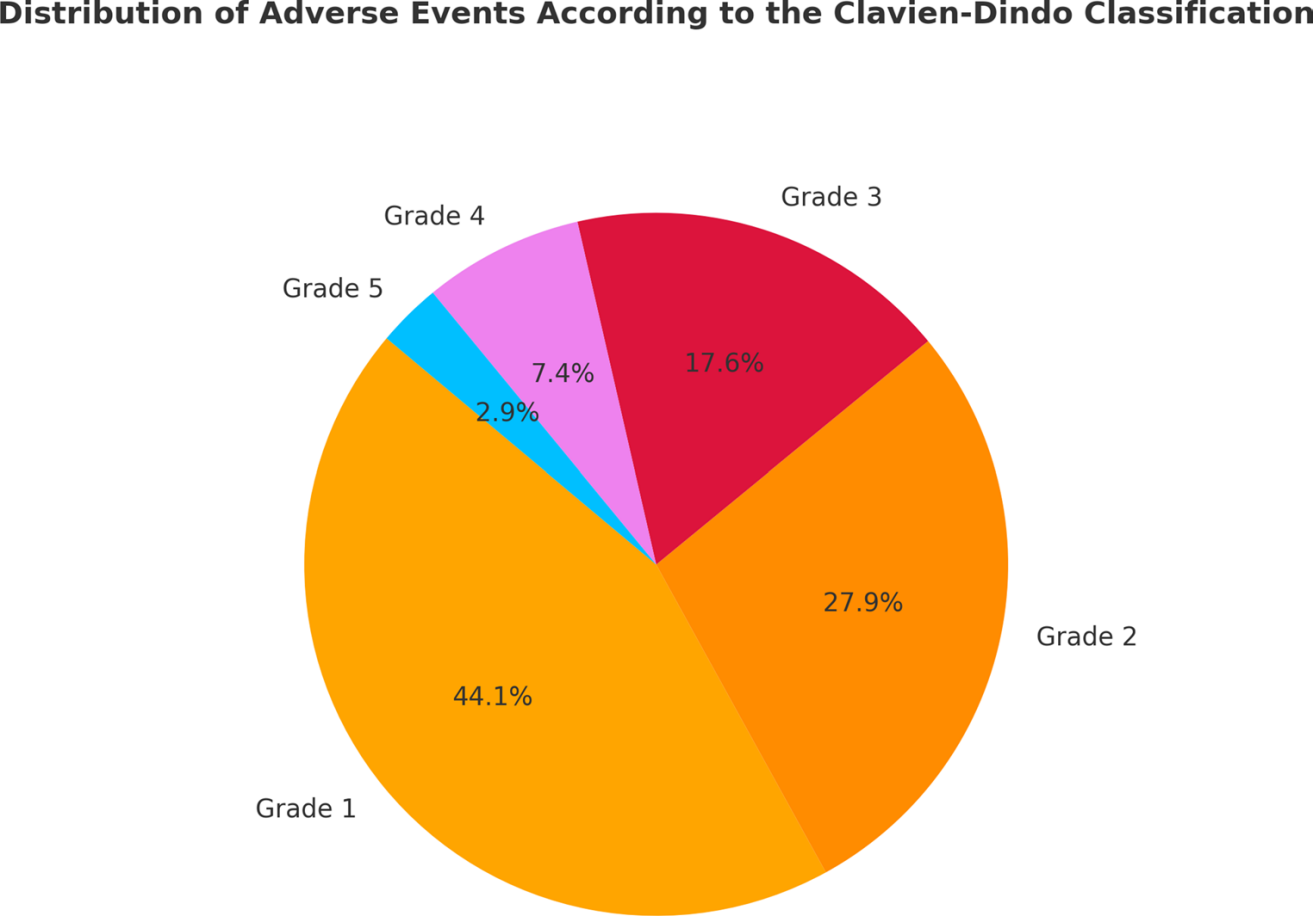
Distribution of AEs according to the Clavien-Dindo classification

Overall, 68 patients (28.8%) experienced at least one adverse event (AE). To facilitate a more structured analysis, we categorized these complications into surgery-related and non-surgery-related AEs. We also assessed how many of these patients required revision surgery due to a complication. According to the Clavien-Dindo classification, 30 patients (12.7%) were assigned to Grade 1, 19 patients (8.0%) to Grade 2, 12 patients (5.0%) to Grade 3, 5 patients (2.1%) to Grade 4, and 2 patients (0.8%) to Grade 5. The distribution of AEs according to the Clavien-Dindo classification is summarized in Fig. 3.

### Surgery-Related adverse events

Surgery-related AEs were observed in 53 patients (22.5%). The most common AE was a new neurological deficit, affecting 33 patients (14.0%). Wound healing disorders occurred in 6 patients (2.5%), postoperative hemorrhage in 6 (2.5%), cerebrospinal fluid (CSF) leakage in 3 (1.3%), seizures in 4 (1.7%), and postoperative hydrocephalus in 1 (0.4%). Overall, 16 patients (6.8%) required revision surgery. Of these, 6 had supratentorial tumors and 10 had infratentorial tumors. Revision procedures were most frequently prompted by postoperative hemorrhage (*n* = 6), followed by wound healing disturbances (*n* = 2), CSF fistula (*n* = 4), and hydrocephalus requiring external ventricular drainage (*n* = 1).

### Non-Surgery-Related adverse events

Non-surgery-related AEs occurred in 20 patients (8.5%). Thromboembolic complications and electrolyte disturbances were each reported in 5 patients (2.1%). Pneumonia and delirium were also observed in 2 patients (0.8%) and 3 Patients (1.3%) respectively. Additionally, Cases of acute kidney injury were observed in 1 patient (0.4%) and urinary tract infection were recorded in 3 patients (1.3%). Other non-specified complications affected 4 patients (1.7%). These results are detailed in Table 2.

**Table 2:** Overview of adverse events

Total Adverse Events (*n*, %)	68/236 (28.8)
Surgery related AEs (n, %)	53 (22.5)
Wound healing disorder	6 (2.5)
CSF Leak	3 (1.3)
Postoperative bleeding	6 (2.5)
New neurological deficit	33 (14.0)
Hydrocephalus	1 (0.4)
Seizure	4 (1.7)
Revision surgery needed (n, %)	16 (6.8)
Supratentorial localization	6 (2.6)
Infratentorial localization	10 (4.2)
Non surgery related AEs (n, %)	20 (8.5)
Thromboembolic event	5 (2.1)
Electrolyte disorder	2 (0.8)
Pneumonia	2 (0.8)
Delir	3 (1.3)
Renal insufficiency	1 (0.4)
Urinary tract infection	3 (1.3)
Others	4 (1.7)

### Risk analysis

In a first-stage analysis, we compared our cohort based on tumor location. We found that surgery-related AEs occurred significantly more frequently in infratentorial tumors (38/106, 35.8%) compared to supratentorial tumors (15/128, 11.7%) (*p* = 0.001). Additionally, patients who underwent surgery via the retrosigmoid craniotomy had a significantly higher incidence of surgery-related AEs compared to those operated on using other craniotomies (34 out of 61 patients, *p* = 0.003). There was no significant difference in surgery related AEs across the ASA grades. However, an ASA score of 3 was more frequently associated with a non-surgery related AE (ASA 1: 1/14, ASA 2: 7/150, ASA 3: 10/41, ASA 4: 0/2; *p* = 0.007). In infratentorial cases, larger tumor diameter was significantly associated with surgery-related AEs (2.8 vs. 2.2 cm; *p* = 0.005), whereas no such association was found in supratentorial tumors (3.2 vs. 3.4 cm; *p* = 0.20). Patient positioning (semi-sitting, park-bench, concorde) did not significantly influence complication or revision rates (*p* = 0.20).

To validate the findings of the univariate analysis, we conducted a binary logistic regression analysis. Risk factors for the occurrence of surgery-related AEs included infratentorial tumor location (OR: 1.23, 95% CI: 1.11–2.51, *p* = 0.001) and surgical duration (OR: 1.01, 95% CI: 1.00–1.01, *p* = 0.005), while maximum tumor diameter, BMI, ASA Score and age were not identified as significant factors. Furthermore, infratentorial tumor location was identified as a unique risk factor for the need for revision surgery (OR: 4.05, 95% CI: 1.02–16.01). For the same adjusted variables, we also performed a risk analysis for the occurrence of non-surgical AEs. Here, age (OR: 1.14, 95% CI: 1.17–1.88, 0.04) and ASA Score (OR: 2.65, 95% CI: 1.08–6.53, *p* = 0.03) showed to be significant predictors. The identified risk factors for the development of AEs and the need for revision surgery are summarized in Table 3.

**Table 3:** Predictors of adverse events and need for revision surgery

	Surgical AE (OR, 95% CI)	*p*-value	Revision surgery (OR, 95% CI)	*p*-value	Non - surgery related AE (OR, 95% CI)	*p*-value
Age	1.01 (0.98–1.04)	0.53	1.47 (0.43–4.97)	0.53	**1.14 (1.17–1.88)**	**0.04**
BMI	1.01 (0.94–1.07)	0.78	1.06 (0.97–1.16)	0.16	1.00 (0.91–1.09)	0.97
ASA	1.54 (0.81–2.93)	0.18	2.18 (0.86–5.48)	0.09	**2.65 (1.08–6.53)**	**0.03**
Tumor diameter in cm	0.96 (0.68–1.35)	0.83	1.31 (0.79–2.15)	0.28	1.20 (0.80–1.77)	0.36
Duration of surgery in min	**1.01 (1.00–1.01)**	**0.005**	1.00 (0.99–1.01)	0.16	0.99 (0.99–1.00)	0.65
Infratentorial localization	**1.23 (1.11–2.51)**	**0.001**	**4.05 (1.02–16.01)**	**0.04**	0.68 (0.52–5.42)	0.38

**Infratentorial Localization**: Reference category = Supratentorial tumors

To further evaluate risk factors for non-surgery related AEs, we performed receiver operating characteristic analyses for age and the ASA score. For non-surgery-related AEs, the AUC for age alone was 0.70 and for ASA score alone 0.72. The multivariable model including both factors yielded an AUC of 0.78, indicating improved discrimination compared with each single predictor.

## Discussion

Complication rates after skull base surgery remain substantial, with retrospective series reporting overall AE rates of 30–55%, depending on surgical approach and case complexity [19]. The most frequent complications include CSF leaks, meningitis, visual impairment, cerebral infarction, and abscess formation [20]. Recent advances such as minimally invasive techniques, refined anatomical knowledge, intraoperative guidance, and multidisciplinary care have improved safety but have not eliminated risk [21]. Against this backdrop, prospective data remain scarce. Our single-center cohort of 236 consecutively treated patients adds to the literature by systematically documenting both surgical and non-surgical AEs within 30 days. By prospectively identifying key risk factors—including infratentorial tumor localization, the retrosigmoid craniotomy, longer operative times, and advanced patient age—our study confirms previously reported associations while providing skull base–specific, context-driven validation.

A salient point of comparison is the retrospective series by Aftahy et al. [7] which reported a revision-requiring adverse event in 17% of 88 patients with midline supratentorial skull base meningiomas. Adverse events that did not require revision surgery were not analyzed. Notably, that study omitted newly developed neurological deficits from its AE definition. In contrast, our prospective study classified any new neurological deficit—such as a new-onset facial nerve palsy—as an AE, capturing a broader range of complications that may not necessitate revision surgery but can significantly impact patient quality of life. Even with this inclusive definition, our overall AE rate (28.5%) and revision surgery rate (6.5%) were comparatively lower, suggesting that a combination of refined operative strategies, meticulous perioperative planning, and rigorous AE tracking protocols may help reduce complication rates. Further insight into postoperative risks is provided by Magill et al. [22], who investigated tuberculum sellae meningiomas in a large retrospective multicenter cohort. Complication rates ranged from 19.9% (transcranial approach) to 31.8% (endoscopic endonasal approach), underscoring the variability in outcomes based on tumor location, approach, and institutional expertise. Although these figures are not directly comparable to our data—given our broader inclusion of various supratentorial and infratentorial tumors—they highlight the importance of individualizing surgical strategies to address the particular characteristics of each lesion.

Our findings also underscore the significance of infratentorial tumor location, which showed a notably higher incidence of surgery-related AEs (35.8%) and revision surgeries (9.4%) compared with supratentorial tumors (11.7% AEs, 4.6% revision rate). These results corroborate large retrospective series such as Samii and Matthies [23] and Falcioni et al. [24], which documented considerable morbidity—particularly cranial nerve deficits, CSF leaks, and vascular complications—when operating in the cerebellopontine angle and petroclival region. For instance, Samii and Matthies described permanent severe facial nerve deficits in approximately 4% of 1,000 vestibular schwannoma patients, while Falcioni et al. emphasized tumor size as a primary predictor of postoperative facial nerve outcomes. Our data similarly highlight the retrosigmoid craniotomy as a key risk factor for surgery-related AEs, likely reflecting the technical complexity of accessing the cerebellopontine angle and the often larger or more intricate lesions encountered in that region.

An important debate in the current literature concerns whether a new cranial nerve palsy—particularly a transient facial nerve palsy—should be classified as an AE. As observed in the studies by Falcioni et al. and Samii and Matthies, transient facial nerve palsy is an expected postoperative outcome following surgeries in the cerebellopontine angle, particularly for vestibular schwannomas, with most cases resolving over time. Our prospective methodology addresses this issue by capturing any new deficit, thereby enabling early detection and intervention while also providing a more comprehensive reflection of actual patient experiences. This inclusivity likely explains why our initial AE rate is higher than in some retrospective reports that focus solely on revision-requiring complications or exclude neurological deficits.

Comparing our results to those of Aftahy et al.[5] - who analyzed 517 patients undergoing retrosigmoid surgery for cerebellopontine angle tumors—we again note methodological and definitional differences. While that study identified a higher revision surgery rate (11.3%) and a slightly lower overall complication rate of 21.1%, its AE criteria emphasized revision surgeries and postoperative facial nerve deterioration. Our prospective AE tracking system, which included both clinical and radiological follow-up to 30 days postoperatively (and beyond for hospital readmissions), may offer a more granular understanding of the full spectrum of complications. Nevertheless, our revision surgery rate (9.4%) for infratentorial tumors was lower, suggesting that detailed, proactive monitoring and standardized surgical protocols can help mitigate severe postoperative complications. In addition to these key factors, we also evaluated tumor size and patient positioning. Tumor diameter was associated with higher complication rates only in infratentorial cases, while positioning did not significantly influence outcomes. Although these findings are not surprising, they illustrate that not all commonly debated intraoperative variables translate into risk differences, and highlight the value of prospective databases that enable the recognition of AEs and help develop strategies to mitigate them.

One potential explanation for higher rates of AEs in infratentorial compared with supratentorial cases in our analysis is likely multifactorial. The posterior fossa represents a narrow corridor with densely packed cranial nerves and critical vascular structures, reducing maneuverability and tolerance for retraction. CSF leaks are more common due to petrous bone air cells and dural closure challenges. Patient positioning may also play a role: the semi-sitting position facilitates venous and CSF drainage but requires vigilance for air embolism, while park-bench positioning limits surgical angles. Finally, the retrosigmoid craniotomy, although a standard workhorse, is often employed for larger or adherent cerebellopontine angle tumors, inherently increasing operative complexity. Together, these factors plausibly explain the higher complication and reoperation rates observed in infratentorial surgery.

A strength of our approach lies in its prospective AE documentation, supported by the Clavien-Dindo classification [11] and routine use of Morbidity and Mortality Conferences (MMCs). By systematically collecting data on all AEs—both surgery-related and non-surgery-related—we were able to track complications from initial treatment until 30 days postoperatively, even capturing those that occurred after discharge in cases of hospital readmission. Additionally, MMCs and departmental review sessions allowed for immediate feedback loops, fostering an environment of continuous quality improvement. Other authors [25, 26] have similarly noted that such structured conferences contribute significantly to reducing recurrence of similar complications and cultivating a learning atmosphere for surgical trainees and the entire neurosurgical team.

Non-surgery-related AEs were observed in 20 patients (8.5%), underscoring the importance of thorough perioperative management to reduce medical complications such as pulmonary embolism, pneumonia, urinary tract infections, and delirium—particularly in older or comorbid patients. Although the overall mortality in our cohort was low (0.8%), both deaths were caused by fulminant pulmonary embolism, highlighting the clinical relevance of VTE in skull base surgery. This complication is especially feared because it may occur abruptly and prove fatal even in patients with histologically benign tumors. Our findings are consistent with retrospective studies that identified immobility, comorbidities, and prolonged operative times as major VTE risk factors in skull base populations [27–29]. More recently, a prospective study demonstrated the feasibility of early screening for venous thrombosis in this setting [30]. Taken together, these data and our results emphasize the need for systematic perioperative VTE risk assessment, appropriate prophylaxis, and—where feasible—early detection protocols in skull base surgery.

Moving forward, larger multicenter prospective studies and subgroup analyses—particularly focusing on elderly patients and those with significant comorbidities—will help refine risk stratification models and inform more precise treatment algorithms. This aligns with a broader trend toward personalized neurosurgical care, wherein the choice of surgical craniotomy, intraoperative adjuncts (e.g., neuromonitoring, endoscopic assistance), and enhanced perioperative recovery protocols are customized to the patient’s specific tumor characteristics and overall clinical context. Overall, our study adds to the growing evidence base that prospective, systematic documentation of complications—combined with continual team-based review and a culture of open, structured feedback—can substantially improve patient outcomes in skull base surgery. By shedding light on the variables that most strongly predict adverse events, we hope to guide neurosurgeons in refining surgical techniques, optimizing patient selection, and implementing robust perioperative care pathways that collectively reduce morbidity and mortality in this challenging field.

### Limitations

Although this study represents a prospective analysis of postoperative adverse events (AEs) in skull base surgery, several important limitations should be noted. First, despite its prospective design, the overall sample size is still modest when stratified according to variables such as supratentorial vs. infratentorial localization and surgical craniotomy, restricting the power for meaningful subgroup comparisons. Second, although a variety of tumor entities and surgical craniotomies were included, direct comparisons with other studies are limited by differences in methodology, patient selection, and definitions of complications. Third, the study focuses primarily on postoperative AEs, precluding an in-depth exploration of pre- and postoperative symptom trajectories, recurrence patterns, and intraoperative anatomic considerations—all of which can be integral to patient outcomes. While broadening the scope to include these elements could enrich the analysis, it might dilute the clarity of the core objective focused on postoperative complications. Additionally, because skull base tumors are relatively rare, extending the study duration and adopting a multicenter approach would likely enhance the statistical power and generalizability of the findings. Moreover, this cohort did not include patients who underwent endonasal endoscopic procedures, a rapidly evolving technique in skull base surgery. Incorporating these cases in future studies would provide a more comprehensive perspective on contemporary surgical practices and outcomes. While we report AUCs for age, ASA score, and their combination, no internal validation (e.g., bootstrapping) or calibration analysis was performed, and AUCs were not statistically compared. These aspects should be addressed in future, larger cohorts to improve generalizability.

## Conclusion

This prospective study provides a systematic analysis of postoperative adverse events (AEs) in skull base surgery, identifying infratentorial tumor localization and prolonged surgical duration as significant risk factors for surgery-related AEs and revision surgery, with the retrosigmoid craniotomy showing the highest complication rate. Advanced age and higher ASA scores were associated with an increased risk of non-surgical AEs, emphasizing the need for comprehensive perioperative assessment. While the overall incidence of complications and revision surgery was low, the findings underscore the importance of careful patient selection, surgical craniotomy optimization, and structured postoperative monitoring. Further multicenter prospective studies with larger cohorts and subgroup analyses, particularly in elderly patients and those undergoing endoscopic procedures, are necessary to refine risk stratification and improve surgical outcomes in skull base surgery.

## Data Availability

The datasets generated and/or analyzed during the current study are available from the corresponding author upon reasonable request.
